# Intraperitoneal Onlay Mesh (IPOM Plus) Repair Versus Extended-View Totally Extraperitoneal Rives-Stoppa (eTEP-RS) Repair in Primary Ventral Hernias: Experience With 50 Cases in a Tertiary Care Hospital

**DOI:** 10.7759/cureus.57678

**Published:** 2024-04-05

**Authors:** Sachin Sholapur, Aftab Shaikh, Abhinav C G, Amarjeet Tandur, Harshal D Padekar, Ajay Bhandarwar, Saurabh Jagdale

**Affiliations:** 1 General Surgery, Grant Government Medical College and Sir JJ Group of Hospitals, Mumbai, IND

**Keywords:** transfascial sutures, primary ventral hernias, laparoscopic ventral hernia repair, rives-stoppa, double crown technique, extended view total extraperitoneal repair (etep-rs), intraperitoneal onlay mesh repair (ipom plus)

## Abstract

Background

Primary ventral hernias are abnormal protrusions of abdominal viscera through the areas of weakness in the fascia of the abdominal wall. The aim of this study was to compare the benefits and complications, and the overall outcome in the Extended-View Totally Extraperitoneal Rives-Stoppa (eTEP-RS) repair versus Intraperitoneal Onlay Mesh (IPOM Plus) repair in the management of primary ventral hernias.

Methods

After obtaining institutional ethical committee clearance, this prospective comparative study between IPOM Plus and eTEP-RS was conducted in a tertiary care hospital from December 2020 to January 2022. A total of 50 patients presenting with primary ventral hernias were included in the study, of whom 25 underwent IPOM Plus and 25 underwent eTEP-RS repairs. Group selection was done by simple randomization using the lottery method.

Patients more than 18 years of age with primary ventral hernias presenting with a hernial defect width less than 6 cm, consenting to the study, were included in the study. Patients who did not fulfill the inclusion criteria, strangulated/obstructed hernias, recurrent/incisional hernias, connective tissue disorders, skin infections, enterocutaneous fistulas, pregnancy, morbid obesity, and parastomal hernias were exclusion factors.

Results

The mean intraoperative duration in the eTEP-RS group (192.3 ± 16.20 min) was significantly higher than in the IPOM Plus group (102.6 ± 16.78min, p=0.001). The mean duration of hospital stay in the IPOM Plus group (5.9 ± 2.19 days) was longer than in the eTEP-RS group (4.6 ± 3.17 days, p=0.02). The mean postoperative pain scores, from the Visual Analogue Scale (VAS), on days 1, 7, and 90 were 3.2 ± 1.11, 2.64 ± 1.11, and 1.68 ± 1.46 in the IPOM Plus group and 1.84 ± 0.688, 0.76 ± 0.66 and 0.08 ± 0.40 in the eTEP-RS group, respectively (p=0.001).

Conclusion

Despite being a technically easy procedure requiring less intraoperative time, IPOM Plus had several disadvantages, such as increased postoperative pain, longer duration of hospital stays, higher chances of wound site seromas, and higher rates of postoperative paralytic ileus*.* On the other hand, eTEP-RS was a more challenging procedure requiring more intraoperative time; however, it had several advantages: less postoperative pain, less duration of hospital stay, early recovery, and fewer chances of seromas and paralytic ileus*.* However, more robust data is required to compare and validate the differences between both procedures' short- and long-term outcomes.

## Introduction

Primary ventral hernias are abnormal protrusions of abdominal viscera through the areas of weakness in the fascia of the abdominal wall [1]. Intra-abdominal pressure exerts its most significant force on the thinnest portion of the wall, leading to progressive widening of the hernia defect if not treated. Conventional ventral hernia repair involves primary defect closure and reinforcement with a prosthetic mesh [2-4]. Laparoscopic repair has been proven to be promising in comparison to open hernia repair, with reduced intraoperative blood loss, postoperative pain, infection, seromas, duration of hospital stay, and intensive care unit (ICU) admissions leading to early recovery, better quality of life and significantly reduced overall hospital costs [5]. Extended-View Totally Extraperitoneal Rives-Stoppa (eTEP-RS) repair and Intraperitoneal Onlay Mesh (IPOM Plus) repair are the two versatile minimally invasive options available in the therapeutic armamentarium of modern ventral hernia repair.

The IPOM Plus repair involves closure of the hernia defect and reinforcement with a prosthetic composite mesh on the peritoneum. To prevent migration, the mesh must be fixed with trans-fascial sutures or absorbable tacks. Though it is a relatively standard procedure performed, several mesh-related complications due to the direct contact of the mesh with the abdominal viscera and postoperative tack-related pain can’t be ignored. To overcome technical difficulties like limited workspace for dissection, restricted trocar set-up, low tolerance to pneumoperitoneum, and poor ergonomics in the traditional TEP repair of challenging inguinal hernias, Jorge Daes modified the procedure to eTEP by approaching the preperitoneal space through the retro-rectus space [6,7]. This was achieved by shifting the camera port more cranially. Igor Belyanksy applied the same technique to repair ventral hernias as eTEP-RS. The eTEP-RS repair is based on the Rives-Stoppa technique, which provides the advantage of completely excluding the mesh from the intraperitoneal domain, where the mesh is placed in a sizeable retro-rectus space without any penetrating fixation. Though this technique provides several advantages over the IPOM Plus repair, the steep learning curve and the prolonged intraoperative duration are matters of concern.

This prospective comparative study was conducted in a tertiary care hospital to compare the various intraoperative, immediate postoperative, and late postoperative parameters and overall benefits and disadvantages of the IPOM Plus and eTEP-RS repairs in primary ventral hernias.

## Materials and methods

After obtaining Institutional Ethical Committee (IEC) clearance (IEC/PG/376/Mar/2021), this prospective comparative study between IPOM Plus and eTEP-RS was conducted in a tertiary care hospital (Department of General Surgery, Grant Government Medical College and Sir JJ Group of Hospitals, Mumbai) from December 2020 to January 2022. A total of 50 patients presenting with primary ventral hernias were included in the study, amongst which 25 underwent the IPOM Plus repair, and 25 underwent the eTEP-RS repair. Group allocation was done by simple randomization using the lottery method.

Inclusion/exclusion criteria

Patients over 18 years of age with primary ventral hernias presenting with a hernial defect width less than 6 cm, consenting to the study, were included. Patients who did not fulfill the inclusion criteria, with strangulated/obstructed hernias, recurrent/incisional hernias, connective tissue disorders, skin infections, enterocutaneous fistulas, pregnancy, morbid obesity, and parastomal hernias were excluded.

Technique

All procedures were performed by surgeons with optimum skills in advanced laparoscopic surgery in a single operation theatre complex under general anesthesia. A single dose of the third-generation cephalosporin antibiotic was given intravenously at the time of induction. According to the patient profile, deep vein thrombosis prophylaxis was instituted.

IPOM Plus

**Figure 1:**
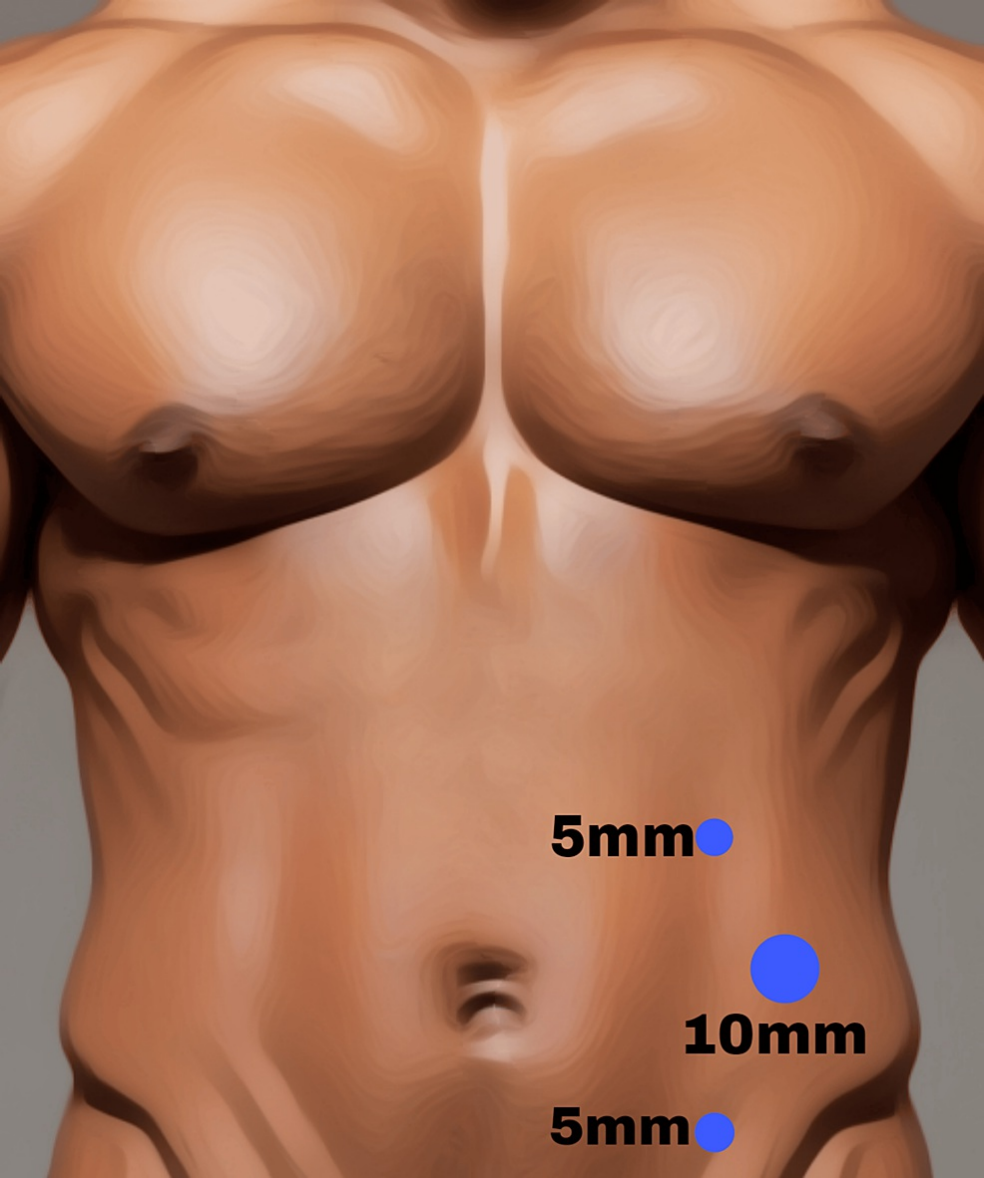
Ports' placement in IPOM Plus IPOM, Intraperitoneal Onlay Mesh Image credits: Author's original work

The patient was placed supine with both arms tucked in to allow adequate space for the surgeon and the assistant on the same side of the table. A Veress needle was inserted at Palmer’s point to create pneumoperitoneum through carbon dioxide insufflation. Most case procedures were performed with lateral ports with a 10-mm camera port placed in the lumbar region in line with the umbilicus just anterior to the midaxillary line. Additional 5-mm working ports were inserted under vision, as shown in Figure 1.

**Figure 2:**
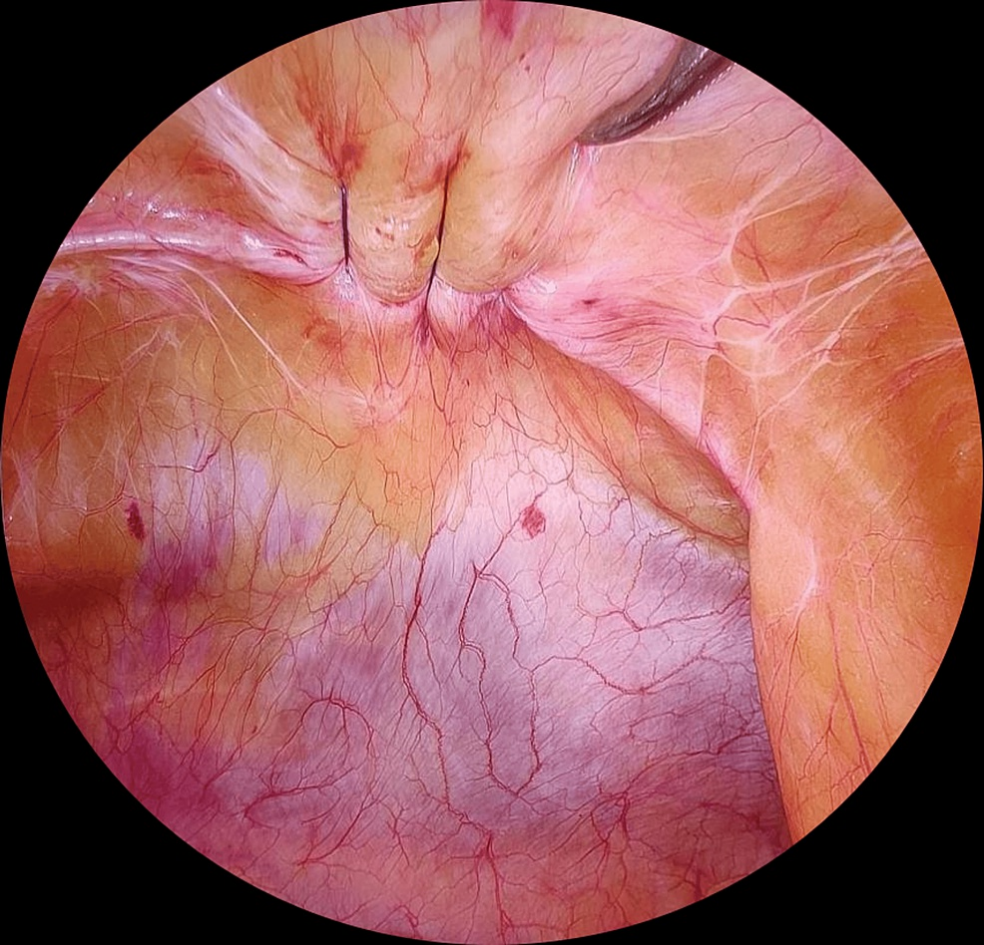
Defect closure using the polypropylene 1 suture Image credits: Author's original work

The pressure was maintained at 12-15 mmHg. A 30-degree telescope was used for all cases. Hernial contents were reduced by applying gentle traction with a grasper and manual pressure from the outside. However, adhesiolysis with cold scissors or ultrasonic shears was needed in some cases with dense adhesions. A polypropylene 1 suture was passed using a suture passer, 1 cm from the fascial edge, to close the defect under vision after reducing the insufflation pressure to 5-8 mmHg (Figure 2).

**Figure 3:**
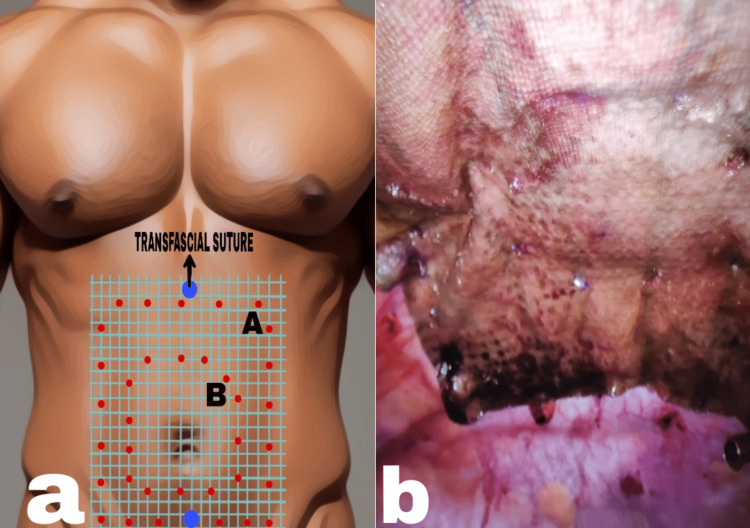
Mesh fixation in IPOM Plus: (a) double crown technique (A, outer; B, inner) and (b) intraoperative picture IPOM, Intraperitoneal Onlay Mesh Image credits: Author's original work

In some cases, the falciform ligament and the bladder flap were taken down to make adequate space for mesh placement. A composite mesh (polypropylene + oxidised regenerated cellulose) of an appropriate size was tailored for a minimum 5-cm overlap in all directions, and two monofilament nonabsorbable sutures were placed at the superior and inferior axial edges of the mesh. The preplaced sutures on the mesh were sequentially withdrawn through preplaced markings on the abdominal wall using a suture passer, and trans-fascial sutures were tied in a subcutaneous plane after reducing the insufflation pressure. We avoided placing trans-fascial sutures at the lateral edges of the mesh to prevent trans-fascial suture site hernias. The mesh perimeter was secured with absorbable tacks placed at 1-cm intervals after tactile perception from the abdominal wall. A second row of tacks was applied at approximately 2-cm intervals and 2 cm from the edge (double crown technique) (Figure 3). The omentum was splayed over the bowel, and the abdomen was deflated under vision.

eTEP-RS

**Figure 4:**
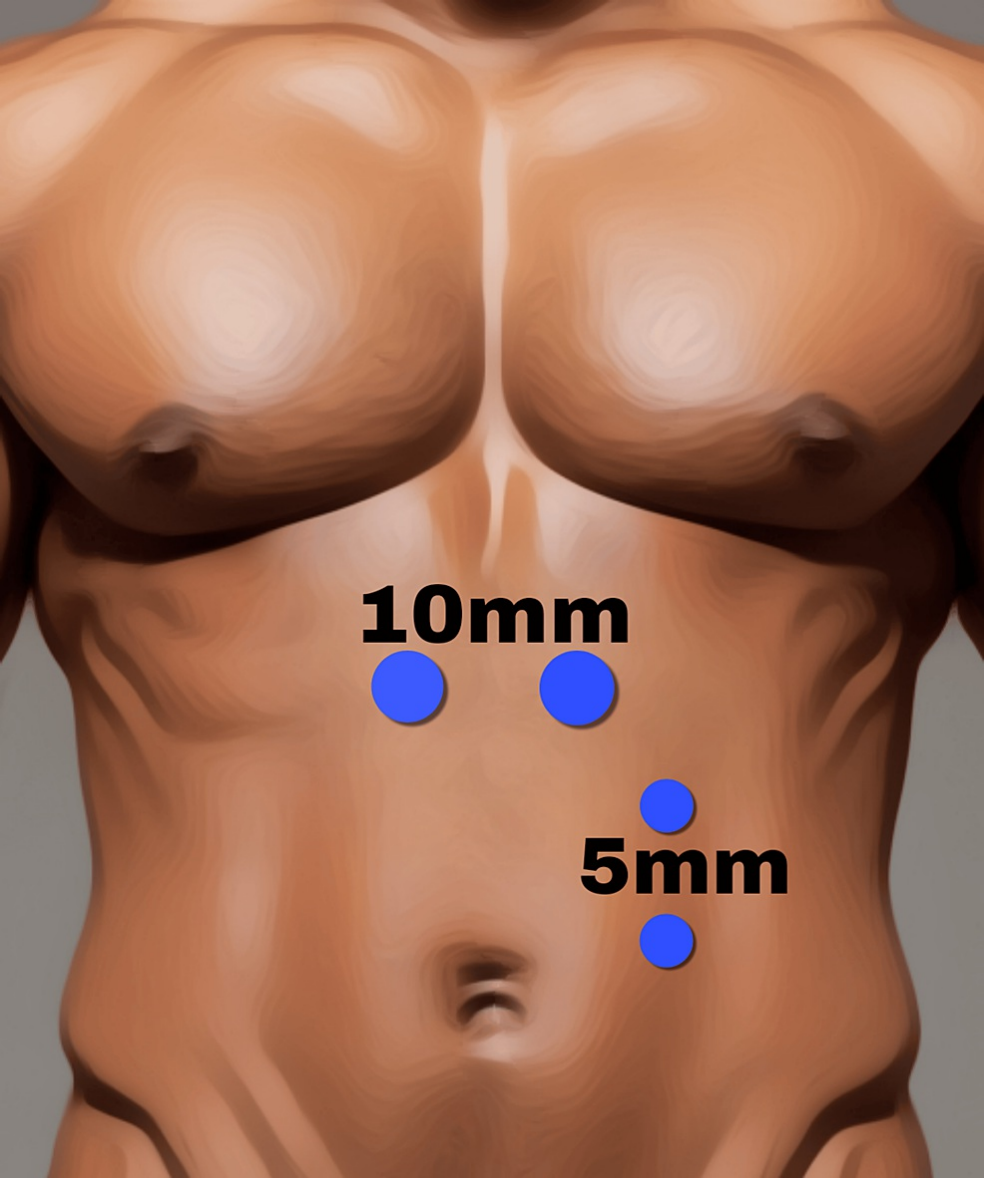
Port placement in eTEP-RS eTEP-RS, Extended-View Totally Extraperitoneal Rives-Stoppa Image credits: Author's original work

The operating table was retroflexed 30-40 degrees after induction of anesthesia to widen the space between the rib margin and the anterior superior iliac crest. The screen was placed at the foot end of the table, and a second monitor was placed on the right-hand side. A 10-mm blunt trocar was placed in the left midclavicular line below the costal margin after incising the anterior rectus sheath and retracting the rectus muscle to identify the posterior rectus sheath (PRS). Left retro-rectus dissection was initially carried out with a zero-degree telescope, and insufflation pressure was maintained at 14 mmHg. Once adequate space was created, the telescope was changed to a 30-degree one, and two more 5-mm working ports were inserted under vision (Figure 4).

**Figure 5:**
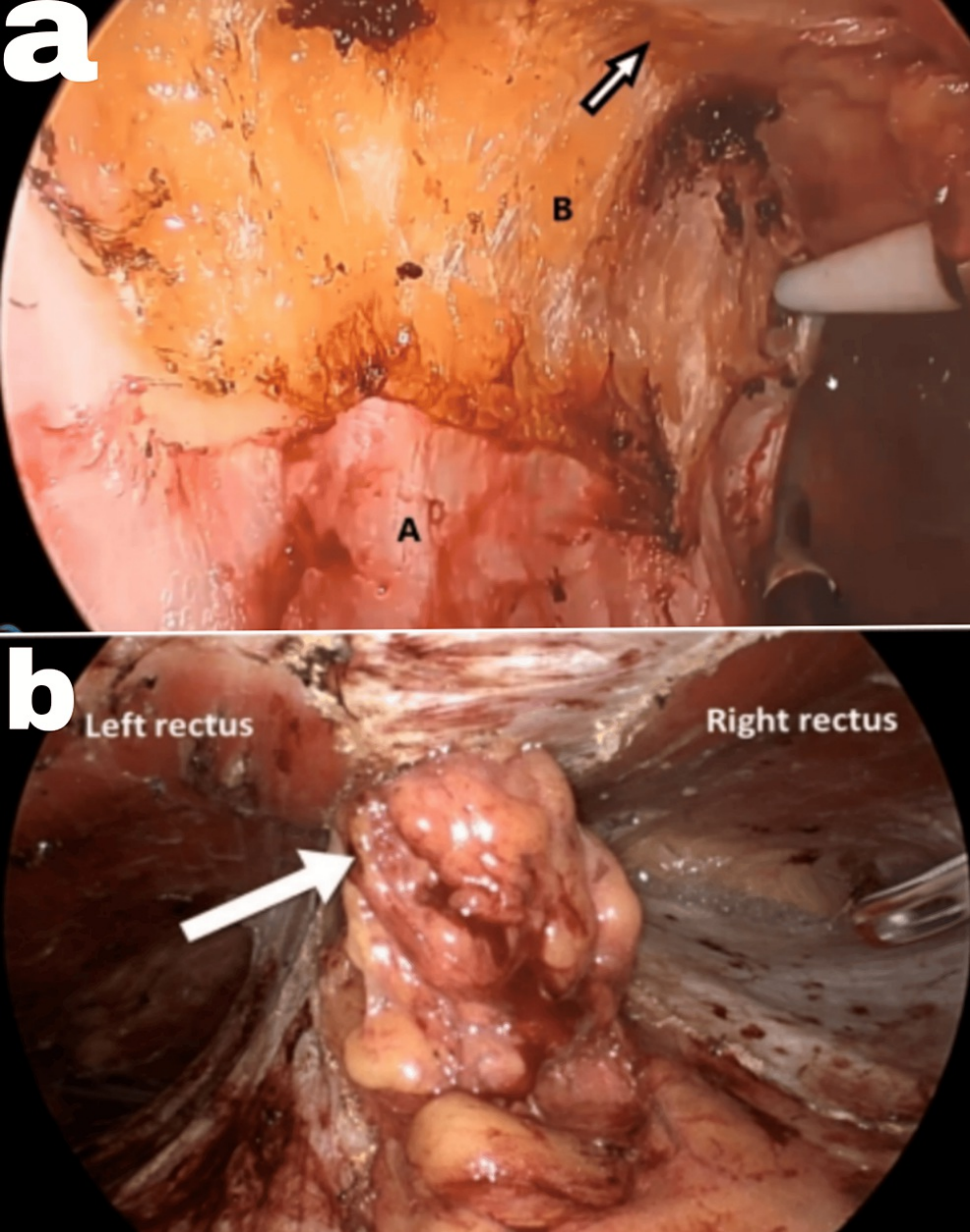
(a) Cranial cross-over (A, posterior rectus sheath; B, fat in the falciform ligament; the white arrow shows the linea alba), (b) a volcano sign with bilateral retro-rectus tunnels Image credits: Author's original work

Dissection was carried out with monopolar diathermy and ultrasonic shears, inferiorly to the Cooper's ligaments and laterally till the neurovascular bundles. By shifting to a 5-mm 30-degree telescope, the cranial cross-over was performed by dividing the left PRS 2-3 mm below the linea alba using a monopolar diathermy hook to visualize the falciform fat (Figure 5a). The falciform was taken down, and the right PRS was divided similarly to access the right retro-rectus space. The right retro-rectus tunnel was developed similarly after inserting an additional 10-mm trocar in the right subcostal area in the midclavicular line. The initial port position was on the left lower abdomen in the upper midline hernias. Dissection was carried out from the caudal to cephalad direction with a caudal cross-over using the pelvic pre-peritoneal spaces. The neurovascular bundles were carefully preserved bilaterally by identifying the 'lamppost sign.' The PRS on both sides was taken down completely in the caudal direction by carefully maintaining the integrity of the linea alba. Once the hernia defect approached, a classical 'volcano sign' was appreciated (Figure 5b).

**Figure 6:**
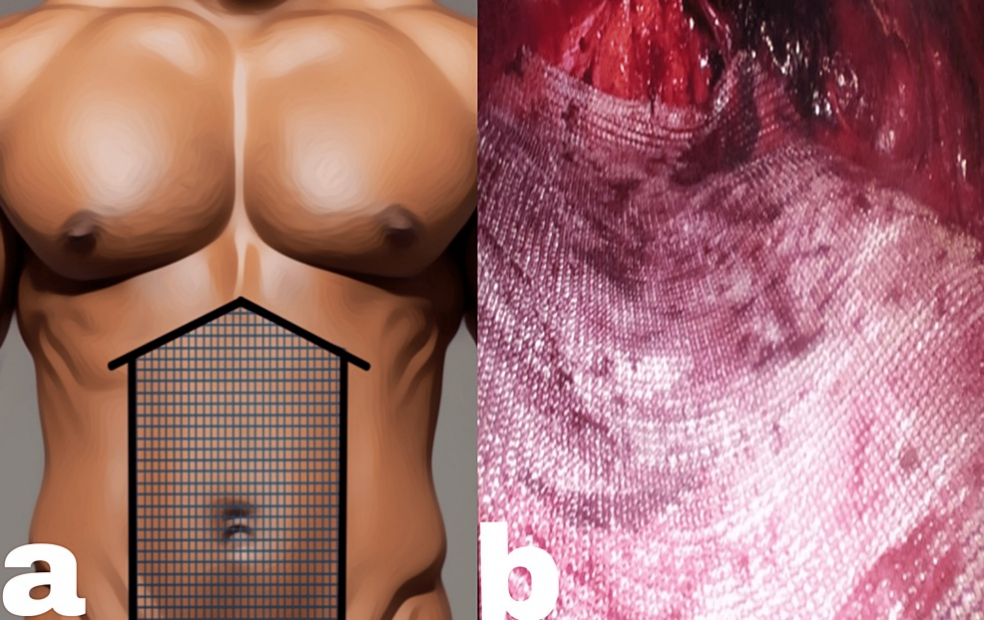
Mesh placement in the eTEP-RS repair procedure: (a) diagrammatic representation of mesh placement, (b) intraoperative picture depicting mesh placement eTEP-RS, Extended-View Totally Extraperitoneal Rives-Stoppa Image credits: Author's original work

The sac with its contents was reduced after adhesiolysis. Once the sac was reduced, the posterior rectus sheaths of both sides were further divided caudally till the pelvic pre-peritoneal spaces were reached. Here, the bilateral retro-rectus spaces meet the pelvic pre-peritoneal spaces, forming one large box where the mesh can be deployed. The hernial defect was continuously closed using barbed sutures like barbed 2-0 sutures. Any rent in the peritoneum was closed using polydioxanone/polyglactin 2-0 interrupted sutures. The divided ends of the posterior rectus sheaths were closed using continuous barbed sutures. A medium-weight macro-porous polypropylene (MWPP) mesh was tailored per the retro-rectus box measurements. The mesh was inserted through the 10-mm port and unrolled to place it in proper orientation to cover the port sites adequately (Figure 6). The mesh was not fixed in any of the cases. CO_2_ desufflation was done under vision.

Statistical analysis

Data was collected, coded, and entered into a Microsoft Excel worksheet (Microsoft Corporation, Redmond, WA), and exported to IBM SPSS software. Data was analyzed using IBM SPSS Statistics, version 21 (IBM Corp., Armonk, NY). Continuous variables were expressed as means ± SD, and categorical variables were expressed as percentages. The chi-square test was used to compare the categorical variables, and a paired t-test was used to compare the continuous variables. A p-value of <0.05 was found to be statistically significant.

## Results

Patients in both groups were statistically comparable in age, sex, body mass index (BMI), and American Society of Anesthesiologists (ASA) class. The mean BMI was 24.2 kg/m^2^ and 25.1 kg/m^2^ among the IPOM Plus and eTEP-RS groups, respectively, with most of the individuals being normal-weight to overweight. Morbid obesity was an exclusion criterion in our study to minimize confounding, as it can be an etiological factor for ventral hernias. Middle-aged adults formed the bulk of our study, with the mean age of patients being 44 and 45 years among the IPOM Plus and eTEP-RS groups, respectively. Most patients had umbilical (15 and 12, in IPOM Plus and eTEP-RS groups, respectively) and paraumbilical hernias (7 and 11, in IPOM Plus and eTEP-RS groups, respectively). The mean defect width in the IPOM Plus group was 2.6 ± 0.91 cm, and in the eTEP-RS group was 3.08 ± 1.30 cm. With a p-value of 0.13, both groups were statistically comparable concerning defect widths. The data is summarized in Table 1.

**Table 1:** Demographic details, presentation and hernia characteristics COPD, chronic obstructive pulmonary disease; BPH, benign prostatic hyperplasia; ASA, American Society of Anesthesiologists; IPOM Plus repair, Intraperitoneal Onlay Mesh (plus stands for defect closure) repair; eTEP-RS repair, Extended-View Totally Extraperitoneal Rives-Stoppa repair

Parameters	IPOM Plus	eTEP-RS
Mean age (years)	44.08 ± 12.05	45.2 ± 10.55
Male	11 (44%)	12 (48%)
Female	14 (66%)	13 (52%)
Mean body mass index (kg/m^2^)	24.23 ± 1.34	25.13 ± 1.78
Comorbidities		
Diabetes mellitus	3 (12%)	4 (16%)
Hypertension	5 (20%)	5 (20%)
Asthma	3 (12%)	1 (4%)
COPD	1 (4%)	0 (0%)
BPH	2 (8%)	1 (4%)
Nicotine use	7 (28%)	6 (24%)
ASA class		
Class I	15 (60%)	14 (56%)
Class II	10 (40%)	11 (44%)
History of previous surgeries	3 (12%)	4 (16%)
Type of hernia		
Umbilical	15 (60%)	12 (48%)
Paraumbilical	7 (28%)	11 (44%)
Epigastric	2 (8%)	2 (8%)
Spigelian	1 (4%)	0 (0%)
Symptoms		
Asymptomatic	8 (32%)	3 (12%)
Progressive bulge	16 (64%)	21 (84%)
Painful bulge	1 (4%)	1 (4%)
Multiple defects	1 (4%)	2 (8%)
Mean defect width (cm)	2.6 ± 0.91	3.08 ± 1.30 (p=0.13)
Mean defect length (cm)	2.01 ± 0.63	2.33 ± 0.94 (p=0.17)
Mean defect area (cm^2^)	5.66 ± 3.64	7.87 ± 5.63 (p=0.11)
Content		
Small bowel	7 (28%)	6 (24%)
Omentum	14 (56%)	11 (44%)
Bowels + omentum	4 (16%)	8 (32%)
Large bowel	0 (0%)	0 (0%)

The mean intraoperative duration in the eTEP-RS group (192.3 ± 16.20 min) was significantly higher than in the IPOM Plus group (102.6 ± 16.78 min, p=0.001). The mean mesh area used in IPOM Plus and eTEP-RS repairs was 201.84 ± 137.89 cm^2^ and 475.92 ± 14.066 cm^2^, respectively. One case in the IPOM Plus group was abandoned due to accidental iatrogenic bowel perforation during adhesiolysis around the sac. One patient in the eTEP-RS group was abandoned due to accidental peritoneal rent during dissection, causing poor maintenance of carbon dioxide insufflation. Two cases in the IPOM Plus group had inadvertent iatrogenic small bowel serosal tears, which were repaired with PDS 3-0 sutures. Various intraoperative parameters are summarized in Table 2.

**Table 2:** Summary of intraoperative parameters CDC, Centers for Disease Control and Prevention; IPOM Plus repair, Intraperitoneal Onlay Mesh (plus stands for defect closure) repair; eTEP-RS repair, Extended-View Totally Extraperitoneal Rives-Stoppa repair; PRS, posterior rectus sheath

Parameters	IPOM Plus	eTEP-RS	p-value
Mean intraoperative duration (min)	102.6 ± 16.78	192.3 ± 16.20	0.001
Intraoperative CDC wound class			-
Clean	24 (96%)	25 (100%)	
Clean contaminated	1 (4%)	0 (0%)	
Anterior reconstruction			-
Barbed 2-0 continuous sutures	-	24 (96%)	
Polypropylene 1 continuous suture	1 (4%)	1 (4%)	
Posterior reconstruction			-
Polypropylene 1 continuous sutures	1 (4%)	1 (4%)	
Polypropylene 1 interrupted sutures	23 (92%)	-	
Barbed 2-0 continuous sutures	1 (4%)	-	
Polydioxanone 2-0 continuous sutures	-	24 (96%)	
Mesh dimensions			-
Mean width (cm)	14.52 ± 5.31	20 ± 3.52	
Mean length (cm)	12.84 ± 4.44	23.8 ± 5.25	
Mean area (cm^2^)	201.84 ± 137.89	475.92 ± 14.066	
Mesh type			-
Medium-weight macro-porous polypropylene (MWPP)	0 (0%)	25 (100%)	
Composite mesh	24 (96%)	0 (0%)	
Mesh fixation type			-
No fixation	0 (0%)	24 (96%)	
Sutured to the abdominal wall + tracker fixation	24 (96%)	0 (0%)	
Sutured to the PRS	0 (0%)	1 (4%)	
No mesh used	1 (4%)	0 (0%)	
Intraoperative complications			-
Major vessel injury	1 (4%)	0 (0%)	
Gastric/bowel injury	3 (12%)	0 (0%)	

The mean duration of hospital stay in the IPOM Plus group (5.9 ± 2.19 days) was longer than in the eTEP-RS group (4.6 ± 3.17 days, p=0.02). The mean postoperative Visual Analogue Scale (VAS) scores on days 1, 7, and 90 were 3.2 ± 1.11, 2.64 ± 1.11, and 1.68 ± 1.46 in the IPOM Plus group and 1.84 ± 0.688, 0.76 ± 0.66, and 0.08 ± 0.40 in the eTEP-RS group, respectively (p=0.001). There was a significant statistical difference between the groups in relation to postoperative pain. All patients were followed up for an average period of three months. None of the patients were missed on follow-up, and none of them died during the period of follow-up. Various postoperative parameters are summarized in Table 3.

**Table 3:** Summary of postoperative parameters IPOM Plus repair, Intraperitoneal Onlay Mesh (plus stands for defect closure) repair; eTEP-RS repair, Extended-View Totally Extraperitoneal Rives-Stoppa repair; VAS, Visual Analogue Scale; UTI, urinary tract infection; SSI, surgical site infection; SSO, surgical site occurrence

Parameters	IPOM Plus	eTEP-RS
Mean total duration of hospital stay (days)	5.9 ± 2.19	4.6 ± 3.17
Drain site		
Subfascial	0 (%)	2 (8%)
Intraperitoneal	2 (8%)	0 (0%)
Subfascial or intraperitoneal with subcutaneous drain	1 (4%)	1 (4%)
No drain placed	22 (88%)	22 (88%)
Postoperative pain		
Mean VAS score on postoperative day 1	3.2 ± 1.11	1.84 ± 0.688
Mean VAS score on postoperative day 7	2.64 ± 1.11	0.76 ± 0.66
Mean VAS score on postoperative day 90	1.68 ± 1.46	0.08 ± 0.40
Postoperative complications		
UTI	0 (0%)	0 (0%)
Paralytic ileus	6 (24%)	1 (4%)
Pneumonia	0 (0%)	0 (0%)
Postoperative SSIs		
Superficial incisional	2 (8%)	1 (4%)
None	23 (92%)	24 (96%)
Postoperative SSOs		
None	13 (52%)	20 (80%)
Skin/soft tissue ischemia	1 (4%)	1 (4%)
Stitch abscess	1 (4%)	1 (4%)
Seroma	9 (36%)	2 (8%)
Infected seroma	0 (0%)	1 (4%)
Wound cellulitis	1 (4%)	0 (0%)
Readmission	1 (4%)	0 (0%)
Reoperation	2 (8%)	0 (0%)
Recurrence	1 (4%)	0 (0%)
Mean days of final follow-up	89.5 ± 3.48 days	88 ± 5.96 days

## Discussion

The Rives-Stoppa sublay mesh repair technique is the gold standard repair technique for ventral hernias [8,9]. It involves placing the prosthetic mesh in the retro-rectus space and reconstructing the linea alba in the midline. IPOM Plus and eTEP-RS repairs are the most commonly performed surgeries in minimal access ventral hernia repairs. However, there is still debate about which of these procedures is superior. Though IPOM Plus is a comparatively simpler and quick procedure to perform, it has a multitude of drawbacks due to the placement of the mesh in the intraperitoneal domain.

On the other hand, eTEP-RS excludes entry into the intra-abdominal domain, where the mesh is placed in the retro-rectus plane, avoiding any contact with the bowel. This comes at the cost of a steep learning curve where the surgeon operates in a narrow space, leading to prolonged intraoperative duration. Many studies have described the relevance of both procedures individually. Surprisingly, more robust studies comparing short-term outcomes of both procedures in a single center are less known in the literature.

The mean intraoperative duration among the IPOM Plus and eTEP-RS groups was 102.6 min and 192.3 min, respectively, with a significant statistical difference (p<0.05). Similar results were found in other comparative studies by Bellido et al., Bui et al., Kumar et al., and Penchev et al. (Table 4) [10-13]. The mean hernia defect width among the IPOM Plus group was 2.6 cm, and in the eTEP-RS group was 3.08 cm. The mean defect area was 5.66 cm^2^ and 7.87 cm^2^ among the IPOM Plus and eTEP-RS groups, respectively. Belyansky et al. noted a gray area between the defect sizes of 6-10 cm where an additional transversus abdominis release (TAR) procedure was required along with eTEP-RS [14]. Hence, only primary ventral hernias with less than 6-cm defects were included in the study.

**Table 4:** Comparison between various studies in terms of the mean intraoperative duration IPOM Plus repair, Intraperitoneal Onlay Mesh (plus stands for defect closure) repair; eTEP-RS repair, Extended-View Totally Extraperitoneal Rives-Stoppa repair

Studies	Groups	Mean intraoperative duration (min)
Our study	IPOM Plus	102.6 ± 16.78
	eTEP-RS	192.3 ± 16.20
		p<0.05
Bellido et al. [10]	IPOM Plus	61.4 ± 18
	eTEP-RS	106.8 ± 20.5
		p<0.05
Bui et al. [11]	IPOM Plus	82.4
	eTEP-RS	103.4
		p=0.01
Kumar et al. [12]	IPOM Plus	75.83 ± 8.35
	eTEP-RS	107.52 ± 23.44
Penchev et al. [13]	IPOM Plus	90 ± 31
	eTEP-RS	186 ± 62
Belyansky et al. [14]	eTEP-RS	218.9 ± 111.2

Mean postoperative VAS scores on days 1, 7, and 90 were comparatively higher in the IPOM Plus group than in the eTEP-RS group. A total of 26% of patients had residual pain after the IPOM Plus procedure in a study by Misiakos et al. [15]. Postoperative pain after a laparoscopic ventral hernia repair depends on the type of mesh fixation [16,17]. Fixing the mesh to the abdominal wall with tacks and trans-fascial sutures is associated with increased postoperative pain and hematoma formation in the IPOM Plus procedure [18]. Entrapment of a nerve during trans-fascial suturing or tacking is known to be the cause of chronic pain. The incidence of chronic pain related to tacker ranges from 1.8% to 28% [19].

On the other hand, the eTEP-RS technique does not require the mesh to be fixed. As the mesh is deployed in a closed retro-muscular space, the mesh migration chances are minimal. The absence of tacks and trans-fascial sutures in eTEP-RS makes it a comparatively painless procedure [20]. Also, the analgesia requirements are comparatively less [21]. A total of 24% of patients in the IPOM Plus group and 4% of patients in the eTEP-RS group experienced postoperative paralytic ileus. The direct contact of mesh with the abdominal viscera is known to cause bowel wall adhesions and resultant paralytic ileus in the postoperative period [22]. A comparison between studies in terms of postoperative parameters has been summarized in Table 5.

**Table 5:** Summary of significant postoperative parameters IPOM Plus repair, Intraperitoneal Onlay Mesh (plus stands for defect closure) repair; eTEP-RS repair, Extended-View Totally Extraperitoneal Rives-Stoppa repair; VAS, Visual Analogue Scale

Studies	Groups	Mean/median total duration of hospital stay (days), p=0.02	Mean VAS score on day 1, p<0.05	Mean VAS score on day 7	Mean VAS score on day 90	Paralytic ileus
Our study	IPOM Plus	5.9 ± 2.19	3.2 ± 1.11	2.64 ± 1.11	1.68 ± 1.46	24%
	eTEP-RS	4.6 ± 3.17	1.84 ± 0.688	0.76 ± 0.66	0.08 ± 0.40	4%
Bellido et al. [10]	IPOM Plus	1.8 ± 0.7	24.4	17.4	2	12%
	eTEP-RS	1.3 ± 0.7	13.8	5.2	1.7	-
		p<0.05	p<0.05	p<0.05	p=0.11	
Bui et al. [11]	IPOM Plus	1	-	-	-	-
	eTEP-RS	0				
		p<0.01				
Kumar et al. [12]	IPOM Plus	1.7 ± 0.66	5.87 ± 0.91	1.63 ± 0.53	-	-
	eTEP-RS	1.11 ± 0.31	2.8 ± 0.62	0.3 ± 0.51		
Penchev et al. [13]	IPOM Plus	3.4	VAS pain score (0-100)	VAS pain score (0-100)	-	-
	eTEP-RS	2.9	IPOM Plus, 38	IPOM Plus, 12		
			eTEP-RS, 11	eTEP-RS, 4		
Belyansky et al. [14]	eTEP-RS	1.0 ± 0.7	-	-	-	-

There were no statistically significant differences between the IPOM Plus and eTEP-RS groups concerning postoperative surgical site infections (SSIs) and surgical site occurrences (SSOs). Local seromas were the most common SSOs found in IPOM Plus (36%) and eTEP-RS (12%) groups. During eTEP-RS, ample space is created in the retro-muscular space, which increases the risk of retro-muscular seroma formation. One such case of retro-muscular seroma was encountered in the eTEP-RS group of our study, which was managed conservatively. Overall, there was no significant statistical difference between the two groups concerning SSOs (p=0.08). A total of 8% of patients in the IPOM group and 4% in the eTEP-RS group developed postoperative local wound infections. There was no significant statistical difference between the two groups concerning SSIs (p=0.32). Percutaneous suturing with multiple sutures may increase the risk of SSIs in IPOM Plus [23]. A summary of these postoperative parameters is provided in Table 6.

**Table 6:** Summary of other relevant postoperative parameters IPOM Plus repair, Intraperitoneal Onlay Mesh (plus stands for defect closure) repair; eTEP-RS repair, Extended-View Totally Extraperitoneal Rives-Stoppa repair

Studies	Groups	Surgical site infections (SSIs)	Seromas	Recurrence
Our study	IPOM Plus	8%	36%	4%
	eTEP-RS	4%	12%	0%
				p=0.32
Bellido et al. [10]	IPOM Plus		35%	2.50%
	eTEP-RS		10.30%	0%
			p=0.01	
Bui et al. [11]	IPOM Plus	2.30%	7%	-
	eTEP-RS	0%	10.30%	
Penchev et al. [13]	IPOM Plus	0%	11.10%	3.70%
	eTEP-RS	0%	14.80%	0%
Belyansky et al. [14]	eTEP-RS	0%	2.50%	0%

The mean total duration of hospital stay in our study was higher in the IPOM Plus (5.9 days) group than in the eTEP-RS (4.6 days) group. This difference was statistically significant at p=0.02. The extended hospital stays in patients undergoing the IPOM Plus repair can be attributed to the higher postoperative pain scores requiring repeated analgesia and higher incidences of paralytic ileus.

Limitations

The current study is a single-center study with a small sample size of 50. Only primary ventral hernias were included in the study, and incisional hernias were not included. A multicentric comparative study with much more robust data is needed to validate the long-and short-term outcomes of IPOM Plus and eTEP-RS in a much more comprehensive way.

## Conclusions

Despite being a technically easy procedure requiring less intraoperative time, IPOM Plus was found to have several disadvantages, such as increased postoperative pain, longer duration of hospital stays, higher chances of wound site seromas, and higher rates of postoperative paralytic ileus. On the other hand, eTEP-RS was found to be a more challenging procedure requiring more intraoperative time; however, it had several advantages, such as less pain in the postoperative period, less duration of hospital stay, early recovery, and fewer chances of seromas and paralytic ileus. However, no statistically significant difference was found between the groups concerning SSIs, SSOs, and recurrence rates. Overall, eTEP-RS was found to be a better procedure for the management of primary ventral hernias than IPOM Plus. However, more robust data is required to compare and validate the differences between both procedures' short- and long-term outcomes.
